# A Novel Empirical Fractional Approach for Modeling the Clogging of Membrane Filtration During Protein Microfiltration

**DOI:** 10.3390/membranes15040099

**Published:** 2025-03-26

**Authors:** Leila Cherifi, Yamina Ammi, Salah Hanini, Mohamed Hentabli, Ouafa Belkacem, Jérôme Harmand

**Affiliations:** 1 Laboratory of Biomaterials and Transport Phenomena (LBMPT), Faculty of Technology, University Yahia Fares of Medea, Medea 26000, Algeria; ammi.yamina@yahoo.fr (Y.A.); hanini.salah@univ-medea.dz (S.H.); hentabli.mohamed@univ-medea.dz (M.H.); belkacem.ouafa@univ-medea.dz (O.B.); 2 Process Engineering Department, Faculty of Technology, Hassiba Ben Bouali University of Chlef, Hay Essalem, P.O. Box 151, Chlef 02000, Algeria; 3 Laboratory of Vegetal Chemistry-Water-Energy, Hassiba Ben Bouali University of Chlef, Hay Essalem, P.O. Box 151, Chlef 02000, Algeria; 4 Laboratory of Environmental Biotechnology (LBE), National Research Institute for Agriculture, Food and the Environment (INRAE), UR0050, University of Montpellier, 11100 Narbonne, France; jerome.harmand@inrae.fr

**Keywords:** membrane filtration, clogging, modeling, dragonfly algorithm, empirical fractional model

## Abstract

This study addresses the pervasive challenge of membrane filtration clogging across various industries. Eight new empirical fractional models are proposed based on the volume accumulation change curve. The effectiveness of these models in predicting material accumulation and characterizing clogging patterns is evaluated. The models are validated against experimental data, achieving impressive coefficients of determination (*R*^2^) between 0.9896 and 0.9997 and relative root mean squared errors (nRMSE) ranging from 0.8674% to 2.9548%. Furthermore, comparing the results with theoretical models of Hermia allows us to relate the empirical models to clogging mechanisms.

## 1. Introduction

Membrane filtration technologies play a vital role in various applications, including producing safe drinking water, treating industrial and domestic waste, filtering beverages, and generating reusable water, and they are crucial in the pharmaceutical industry, ensuring the quality of final products. Additionally, membrane filtration is widely used in biotechnology purification processes to remove bacteria and, increasingly, viruses from fermentation media, buffers, and process streams, enhancing safety and purity [1,2,3,4,5,6]. However, one of the primary challenges encountered in the application of membrane filtration is membrane clogging, which significantly impacts filtration efficiency. This clogging can occur due to various factors, including suspended solids, organic matter, microbial growth, and scaling agents present in the feed solution [4,7]. The clogging process involves the deposition of small particles on the walls of the membrane pores, resulting in a decrease in the available flow area and increased resistance to filtration. Additionally, larger particles can entirely block the pores, exacerbating the fouling problem. Furthermore, as the fouling layer develops, it becomes continuous, reducing filtration performance [2]. Various strategies are employed to mitigate membrane clogging. These include the pre-treatment of feed solutions to remove or reduce fouling agents, the optimization of operating conditions, and the use of cleaning protocols to maintain membrane performance [8]. However, these measures contribute to a subsequent increase in operating costs [9,10]. Researchers and engineers continuously explore innovative approaches, including surface modifications and advanced membrane designs, to enhance resistance against fouling and improve overall filtration efficiency [11]. One of the main problems we encounter is our ability to predict the clogging modes in progress. In this respect, it has been shown that clogging modeling can not only help to detect the occurrence of clogging but also classify it [12,13]. Mathematical models can be empirical, based on experimental data correlation, or phenomenological, rooted in physics. Empirical models lack direct physical interpretation, making them useful when understanding underlying mechanisms is limited. Thus, empirical models may be preferable in such cases due to their flexibility in accommodating data without requiring detailed physical insights [14]. Furthermore, semi-empirical models integrate elements from both empirical and phenomenological modeling approaches [14]. These models correlate observed data with physical principles, balancing empirical accuracy and a deeper comprehension of the underlying processes [14]. Hermia’s work is groundbreaking, presenting a unified clogging model able to describe clogging dynamics. This model derived four standard clogging mechanisms [12] depending on how the solute becomes deposited across the membrane [7,9]. Classical blocking models assume that clogging is directly related to the volume of liquid that is filtered, not only the rate at which filtration occurs, and the relationship between the flow rate and membrane capacity can be explained by the fact that higher flow rates shorten the time foulants spend in the membrane, thereby reducing foulant deposition and increasing membrane capacity [3]. Each of these four mechanisms has been individually used to describe an experimental observation, involving complete clogging, intermediate clogging, standard clogging, and cake formation [3,4,12,15,16,17]. Although many previously developed models have proven their effectiveness, they still have some limitations. A primary limitation is their inability to account for the phenomenon where membrane capacitance can increase with an increasing flow rate [3,18,19]. When other researchers consider the filtration process as complex and consider individual models incapable of elucidating the locations of particle deposits on the membrane, combination models have been proposed to simulate the real situation of the clogging of membrane filtration [19,20,21,22,23,24].

Hlavacek and Bouchet applied intermediate, complete, and standard models to data on the fouling of microporous track-etched membranes (polycarbonate, cellulose, and polyvinylidene fluoride) by BSA, with the intermediate model yielding the best fit [25]. Tracey and Davis initially fit data for BSA fouling using either the standard or complete model, later transitioning to the cake model [26]. Bowen et al. observed flux decline not following individual fouling models, suggesting a combination of complete blocking, standard blocking, and cake formation [18]. Recent models consider the sequential effects of complete clogging and cake formation, as well as the simultaneous effects of two classical fouling mechanisms. Bolton found that incorporating the cake-adsorptive model provided a better fit than the intermediate adsorption model [24].

The study by Affaro Affandy et al. investigates contamination mechanisms in DNA fouling of plasmids pQR150 (20 kb) and pGEc47 (56 kb) during constant pressure filtration within a 0.22 mm PVDF membrane. The analysis, utilizing classical and composite clogging models, discloses a transition between fouling mechanisms. Initially, particle deposition dominates, as captured by the intermediate clogging model, while later stages reveal internal contamination, where the standard clogging model prevails. This study underscores the intricate relationship between plasmid DNA flexibility, membrane structure, and fouling behavior during septic filtration [27]. Many other studies using Hermia’s original models to relate clogging mechanisms to observed data have been reported [13].

This study aims to improve the modeling and understanding of membrane clogging mechanisms and volume accumulation in filtration by utilizing empirical fractional estimations that are evaluated by statistical error criteria. The work is divided into two parts. The first part proposes eight empirical fractional equations to model the clogging of membrane filtration, which is optimized using the dragonfly algorithm. These equations use the accumulated volume as a function of time. The second part focuses on identifying the clogging mechanism in membrane filtration for the given datasets using the general Hermia equation and the proposed models.

## 2. Materials and Methods

### 2.1. Clogging of Membrane Filtration

Membrane microfiltration is utilized for purifying pharmaceuticals and biotech products by removing impurities. Protein fouling can impact the efficiency of the process by causing irreversible changes to the membrane. Clogging in such membrane systems, resulting from material accumulation on membrane surfaces, leads to a progressive reduction in permeation flux over time, especially in systems with constant transmembrane pressure. This decrease in flow has significant consequences, such as a reduced lifespan of the membrane, higher maintenance requirements, increased chemical usage, and extra energy consumption. Cake formation due to clogging creates flow resistance and compromises separation efficiency by adding an extra layer to the original membrane [11]. The classical theory of membrane filtration suggests that fouling can be attributed to one of four primary mechanisms: complete blocking, intermediate blocking, standard blocking, or cake filtration [12]. Complete blocking occurs when particles larger than or comparable to the pore size accumulate at pore entrances and eventually seal them off entirely, progressively reducing the number of functional pores until filtration is no longer possible [3]. Intermediate blocking, a transitional stage between complete blocking and cake filtration, involves the partial obstruction of membrane pores by some particles while others accumulate on the deposited layer, leading to increased hydraulic resistance and a gradual decline in available pores. Standard blocking occurs when fine particles infiltrate the membrane structure and adhere to the internal pore walls, progressively narrowing the pore diameter and reducing permeability over time. Conversely, cake filtration results from the accumulation of particles on the membrane surface, forming a porous cake layer that impedes fluid flow [3,20]. The structural composition of this layer depends on particle size: larger particles create a more permeable deposit, whereas smaller particles form a denser, less permeable layer. As the cake thickens, fluid transport becomes increasingly difficult, ultimately reducing filtration efficiency [17,22]. The filtration behavior given by these models can all be described using the following single mathematical form involving the derivatives of the filtration time (*t*) for the cumulative volume (*v*) [11,17,22,23].

(1) $$\frac{d^{2}t}{{dv}^{2}}=k{\left(\frac{dt}{dv}\right)}^{N}$$

**Figure 1:**
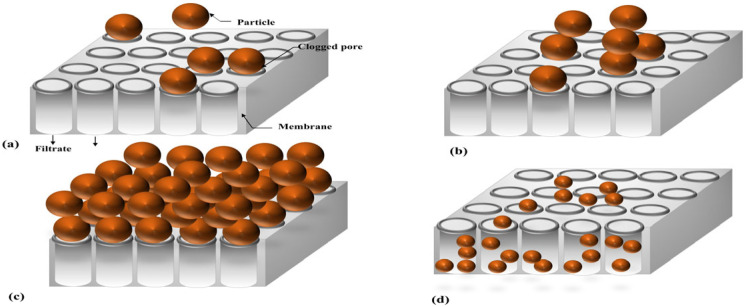
Schematic view of four fouling patterns in blocking filtration: (**a**) complete blocking, (**b**) intermediate blocking, (**c**) cake filtration, and (**d**) standard blocking Reprinted from [22].

Parameter *k* in Equation (1) is the filtration constant. Exponent *N* characterizes the fouling mechanism; it can be shown that *N* = 0 corresponds to cake filtration, *N* = 1 represents intermediate blocking, *N* = 1.5 represents standard blocking, and *N* = 2 represents complete blocking [12]. Figure 1 presents a schematic view of the four filtration clogging mechanisms: (a) complete blocking, (b) intermediate blocking, (c) cake filtration, and (d) standard blocking.

### 2.2. The Proposed Empirical Fractional Estimation Models

In this study, the proposed models are empirical fractional estimation models, directly inspired by the analysis of experimental data collected on the accumulation of retentate in membrane filtration systems over time. The accumulation volume was measured at various time intervals, allowing us to discern distinct patterns in how the membrane is clogged over time. These models are specifically formulated based on these observed patterns rather than derived from pre-existing theoretical models and thus from physical laws. They are designed to accurately describe the dynamic behavior of membrane clogging as reflected in the experimental data. Our models differ from classical ones, like Hermia’s equations, in approach and flexibility. While classical models classify fouling into predefined mechanisms based on physical assumptions [4], our models predict the accumulated volume of retained material over time without directly categorizing clogging mechanisms. This data-driven approach enhances adaptability, as classical models may struggle with complex or evolving clogging behaviors [17]. Our models improve clogging forecasts and filtration optimization by prioritizing predictive accuracy over mechanistic interpretation. Additionally, they estimate the critical accumulation volume leading to complete pore blockage, enabling operators to prevent unexpected shutdowns and extend membrane lifespan. By emphasizing real-world data over theoretical constraints, our models are practical tools for assessing membrane performance in diverse conditions. The empirical fractional estimation models proposed for the clogging of membrane filtration are listed in Table 1. Table S1 (cf. Supplementary Data) presents the units of parameters for each proposed empirical fractional estimation model.

### 2.3. Building a Database

In this study, we utilized data available in the literature from two references [3,27]. The dataset employed for validating the proposed empirical fractional estimation of the clogging of membrane filtration models was extracted from these papers using Get Data Graph Digitizer 2.24. The entire database collected from the literature was divided into four parts according to the different experimental conditions encountered. The first database (DB 01) describes the filtration of 1.0 kg·m^−3^ bovine serum albumin (BSA) through 0.2 m track-etched polycarbonate membranes [3], and the second database (DB 02) defines the filtration of a solution containing 50 mg/mL of two large plasmids (DNA pQR150 (kanamycin resistance 20 kb) and pGEc47 (tetracycline resistance 56 kb)) during constant pressure filtration with a 0.22 mm PVDF membrane at transmembrane pressures of 5 and 8 [27]. While these datasets provide valuable insights into clogging behavior, their diversity remains limited. However, the proposed models are formulated to be applicable beyond the studied conditions. This aspect was acknowledged in the discussion, emphasizing the need for further evaluation with additional datasets. Tables S2 and S3 in the Supplementary Data provide detailed information about the database, with Table S2 focusing on the statistical analysis of the variables and Table S3 focusing on the sizes and various pressures of databases. The graphics program used to plot the fitting is Originlab 2021.

### 2.4. Dragonfly Algorithm Nonlinear Regression (DA_Nlinfit)

**Figure 2:**
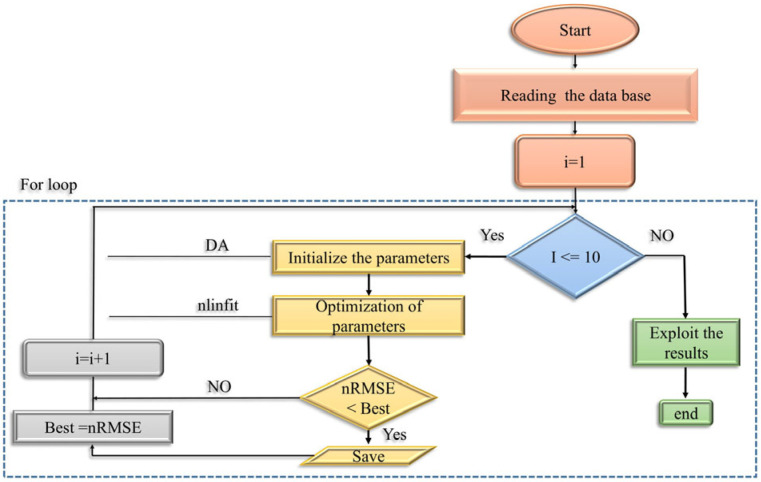
The procedure of the optimization algorithm (Dragonfly Algorithm Nonlinear Regression (DA_nlinfit)).

To identify unknown model parameters, the dragonfly optimization algorithm (DA) was used. It is a flexible optimization method with several advantages. It can operate with minimal controlling parameters and has been applied in various domains, including both research and industrial applications [1,28]. The curve fitting technique is a method for studying experimental curves. It involves constructing a curve using mathematical functions and adjusting the parameters of these functions to approximate the measured experimental curve. So, we also discuss the adjustment parameters [1]. The least squares technique (nlinfit in MATLAB) requires initial values for the model coefficients. The more carefully the initial values are selected, the faster and more directly the technique converges to a solution. Initial parameter values were obtained using the DA, and optimization was performed 10 times. The set of proposed empirical fractional estimation model parameters with the best fit was saved and used as an initial approximation in the nlinfit function with the Levenberg–Marquardt algorithm used as the optimization option [1]. In both cases, the relative root mean squared error (nRMSE) value was used as an objective function to be minimized. Figure 2 presents a summary of the modeling steps.

### 2.5. Performance Study of Proposed Models

The optimal parameter values were obtained by minimizing the objective function involving both experimental and computed values. Each empirical fractional estimation model was applied to all pressure filtrations; the database includes volume versus time data for the first and second databases (DB 01 and DB 02). The performance of each empirical fractional estimation model was evaluated in terms of the Sum of Squares of Residuals (SSR) and relative root mean squared error (nRMSE), together with other statistical error indices (the coefficient of determination (*R*^2^), the variance accounted for metric (VAF), relative mean absolute error (nMAE), relative Chi-square distribution (nChi), and standard error of prediction (SEP)) [1,29,30].

The formulas of error indices used to evaluate the efficacy of the proposed models are shown in Equations (2a)–(2g). Generally, a value of $R^{2}$ greater than 0.5 is considered satisfactory, while a value greater than 0.9 is excellent [31]. Excellent model accuracy is defined as nRMSE < 10%, good accuracy as 10% < nRMSE < 20%, fair accuracy as 20% < nRMSE < 30%, and low accuracy as nRMSE > 30% [30]. All of these indices were programmed and calculated using MATLAB R2021b software.

(2a) $$SSR={\sum_{i=1}^{M}\left(y_{exp.i}-y_{cal.i}\right)}^{2}$$

(2b) $$R^{2}=1-\frac{SSR}{{\sum_{i=1}^{M}\left(y_{exp.i}-\bar{y_{exp.i}}\right)}^{2}}$$

(2c) $$VAF=\left[1-\frac{var(y_{exp.i}-y_{cal.i})}{var(y_{exp.i})}\right]\cdot 100$$

(2d) $$RMSE=\sqrt{\frac{SSR}{M}};\;nRMSE=\left[\frac{RMSE}{\sum_{I=1}^{M}\left(\frac{y_{cal.i}}{M}\right)}\right]\cdot 100$$

(2e) $$MAE=\frac{1}{M}\sum_{i=1}^{M}\left(y_{exp.i}-y_{cal.i}\right);\;nMAE=\left[\frac{MAE}{\sum_{I=1}^{M}\left(\frac{y_{cal.i}}{M}\right)}\right]\cdot 100$$

(2f) $$Chi=\frac{SSR}{\left(M-dim\right)};\;nChi=\left[\frac{chi}{\sum_{I=1}^{M}\left(\frac{y_{cal.i}}{M}\right)}\right]\cdot 100$$

(2g) $$SEP\left(\%\right)=\left(\frac{RMSE}{y_{e}}\right)\cdot 100$$

where *M* is the number of data points; $y_{exp.i}$ and $y_{cal.i}$ refer to the experimental values and calculated values obtained from the proposed model, respectively; $y_{e}$ is the average value of experimental data; and var is the variance.

### 2.6. Identifiability of Model Parameters

Given a model, a criterion, and a dataset, identifiability refers to our capacity to identify a unique set of parameters that minimizes the criterion whatever the initial conditions on the parameters [32,33,34]. There are two distinct notions of identifiability: structural (does the structure of the system yield identifiability?) and practical (do the available data concerning both their quantity and quality—allow for the identification of a unique set of optimal parameters?).

Some of the models, like model 5, are not identifiable since it is over-parametrized: whatever the initial conditions of the parameters within the optimization procedure used to identify their optimal values, d × a^m^ should be constant. We did not conduct specific identifiability in this study because the considered model is much more of a black-box model where parameters do not have any physical meaning; however, white/gray models with parameters are only optimized such that the corresponding models reproduce adequate experimental data.

## 3. Results and Discussion

The adjustment parameters and error values of the proposed empirical fractional estimation models for the clogging of membrane filtration under various pressures (2 psi, 5 psi, 8 psi, 10 psi, and 20 psi) from two databases (DB 01 and DB 02) are detailed in the Supplementary Data, Table S4. This data review demonstrates that the models perform robustly across different pressure and filtration solution conditions, highlighting their reliability and efficiency. Notably, model 5 exhibits the lowest errors among the proposed models across the pressure ranges, with the relative root mean squared error (nRMSE) ranging from 0.8674% to 2.2959%, the relative mean absolute error (nMAE) ranging from 0.6229% to 1.8938%, the standard error of prediction (SEP) ranging from 0.8764% to 2.2954%, and the relative chi-square (nchi) value ranging from 0.0315% to 0.1792%. Among the proposed models, the fifth model exhibited the highest predictive accuracy across various operating conditions. This superiority can be attributed to two key factors. First, the model incorporates more parameters, enhancing its flexibility in capturing variations in clogging behavior and allowing for a more precise fit to experimental data. Second, it effectively accounts for the nonlinear nature of clogging dynamics, improving the accuracy of accumulation trend estimations over time. Other models display lower errors under specific pressure conditions. Furthermore, both the coefficient of determination (*R*^2^) and the variance accounted for metric (VAF) exceed 0.99 and 99%, respectively, underscoring the superior performance of these models. For instance, model 6 shows *R*^2^ values between 0.9936 and 0.9997 and VAF values ranging from 99.3558% to 99.9655%. Overall, the analysis confirms the models’ effectiveness in characterizing the clogging of membrane filtration across various pressures and solutions.

**Figure 3:**
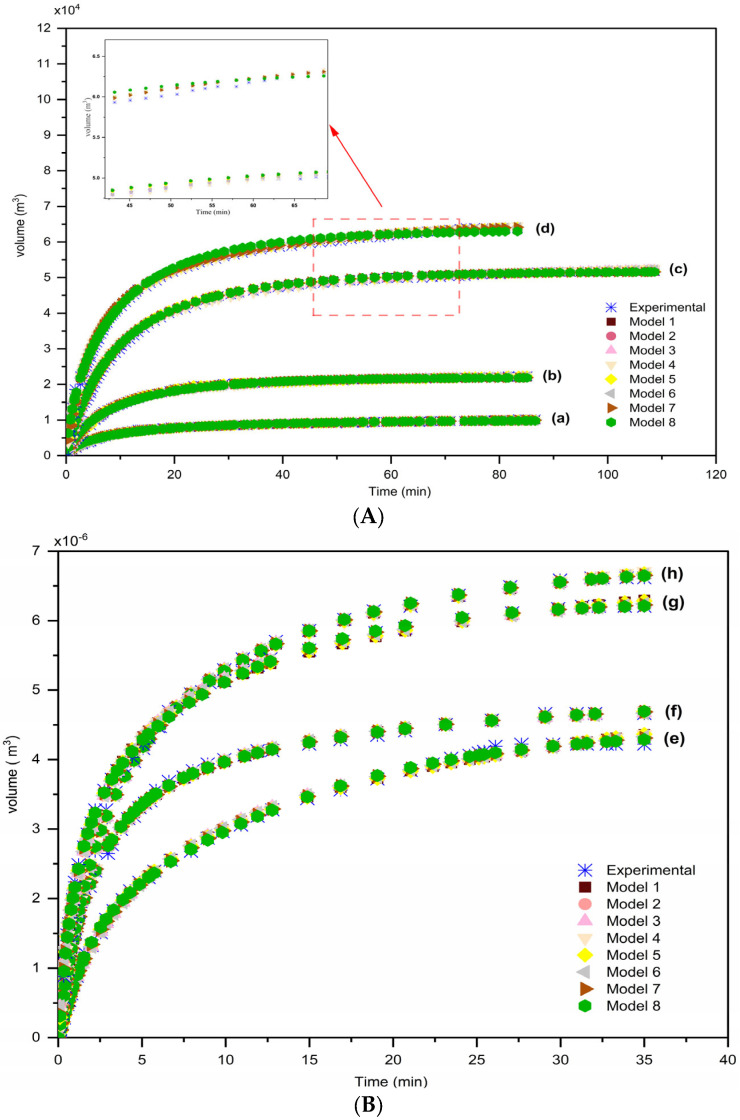
Comparison between calculated and experimental volumes against time for eight models proposed at different pressure filtrations: (a) *P* = 2 psi, (b) *P* = 5 psi, (c) *P* = 10 psi, and (d) *P* = 20 psi for (**A**) DB 01 and (e) *P* = 5 psi (pQR150), (f) *P* = 8 psi (pQR150), (g) *P* = 5 psi (pGEc47), and (h) *P* = 5 psi (pGEc47) for (**B**) DB 02.

Figure 3 shows a comparison between calculated and experimental volumes against time for eight models proposed at different pressure filtrations: (a) *P* = 2psi, (b) *P* = 5psi, (c) *P* = 10 psi, and (d) *P* = 20 psi for DB 01 and (e) *P* = 5 psi (pQR150), (f) *P* = 8 psi (pQR150), (g) *P* = 5 psi (pGEc47) and (h) *P* = 5 psi (pGEc47) for DB 02. The filtrate volume reached its maximum before the full 85 min for DB 01 and at 35 min for DB 02. Generally, infinite volumes $v_{\infty }$ are as follows: $1.0173\times 10^{4}m^{3}$ (2 psi), $2.2283\times 10^{4}m^{3}$ (5 psi), $5.1833\times 10^{4}m^{3}$ (10 psi), and $6.3944\times 10^{4}m^{3}$ (20 psi) for DB 01 and $4.2216\times 10^{-6}m^{3}$ (5 psi pQR150), $4.6639\times 10^{-6}m^{3}$ (8 psi pQR150), $6.6177\times 10^{-6}m^{3}$ (5 psi pGEc47), and $6.2080\times 10^{-6}m^{3}$ (8 psi pGEc47) for DB 02. The infinite volume values indicate pore clogging and filtration cessation. The analysis confirms that the proposed empirical fractional estimation models accurately predict filtrate volume across different pressure conditions. The strong agreement between calculated and experimental values demonstrates their reliability in characterizing membrane clogging dynamics and predicting filtration behavior under varying conditions. Since the experimental datasets cover short filtration durations (85 min for DB 01 and 35 min for DB 02), the models are well suited for capturing early clogging dynamics, where significant flux variations occur. Although evaluated on short-term data, the models are not inherently limited to these conditions and can be applied to longer filtration durations if such data become available. Future studies could explore an adaptive modeling approach with periodic parameter updates to improve predictive accuracy over extended filtration periods.

Table S5 in the Supplementary Data presents the linear regression vector values derived from comparing the calculated and experimental volumes, as shown in Figure S1 (c.f. Supplementary Data). Additionally, it provides performance metrics for the eight proposed empirical fractional estimation models. The results highlight the robustness of these models, with regression vectors closely aligning with ideal values (α = 1 for slope, β = 0 for intercept, and R = 1 for correlation coefficient). Each model was rigorously evaluated across varying pressures and solutions, demonstrating consistent and reliable performance under different filtration conditions.

**Figure 4:**
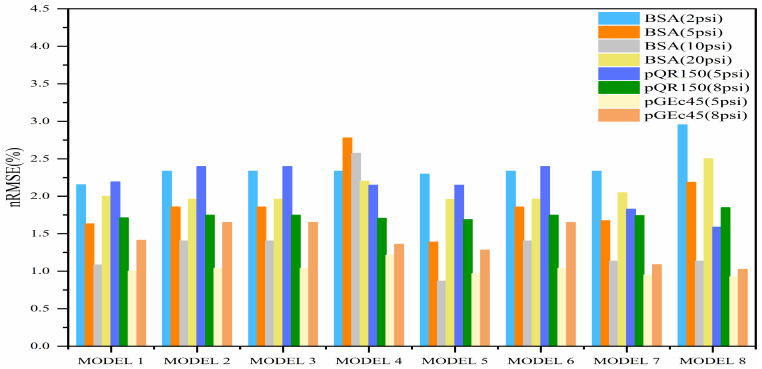
nRMSE plot comparison of proposed models for different pressures and solutions.

Figure 4 shows another comparison between the proposed models at different pressures and solutions in terms of the relative root mean squared error (nRMSE). The proposed models demonstrated a positive outcome with the nRMSE values, showcasing the capability and flexibility of empirical fractional estimation models in modeling the clogging of membrane filtration at different pressures and solution conditions. Remarkably, the fifth model stood out by yielding optimal nRMSE values compared to the other models. All comparisons and results from the previous analyses affirm the effectiveness of the models in predicting the profiles of the clogging of membrane filtration.

### An Analysis of the Clogging of Membrane Filtration Mechanism Based on the Equation of Hermia

Understanding the fouling mechanism is crucial for determining the ideal filtration conditions, including process parameters, interactions between the product and the membrane, and optimizing various membrane characteristics like the pore structure [27,35]. To understand the process of clogging of membrane filtration, a mathematical analysis was performed to investigate the transition of the clogging mechanism. The coefficient of the equation of *N* (clogging index) was obtained by taking logarithms on both sides of the equation of Hermia (1) followed by a linear regression to yield the following form:

(3) $$ln\left(\frac{d^{2}t}{{dv}^{2}}\right)=N\cdot ln\left(\frac{dt}{dv}\right)+C$$

where *C* is the constant ($C=ln\left(k\right);k=exp(C)$) and *k* presents the constant of clogging.

However, it should be noted that the effectiveness of this analysis, i.e., the statistical precision of the values of N, is strongly dependent on the goodness of fit of the expressions and the original volume–time data. The derivatives of $\left(\frac{dt}{dV}\right)$ and $\left(\frac{d^{2}t}{{dv}^{2}}\right)$ were computed by taking the analytical derivatives of model 6 (model 6 gives a good fit and has the fewest constants). It is worth mentioning that small discrepancies between the volume–time data and the best-fit curve could amplify errors between the actual derivatives of $\left(\frac{dt}{dV}\right)$ and $\left(\frac{d^{2}t}{{dv}^{2}}\right)$. As our interest lies in the change in the general law constants, it is possible to control such errors to some extent by repeating the best-fit analysis on subsets of the data. Table 2 presents equations of model 6, its derivatives, and the equation of Hermia.

**Figure 5:**
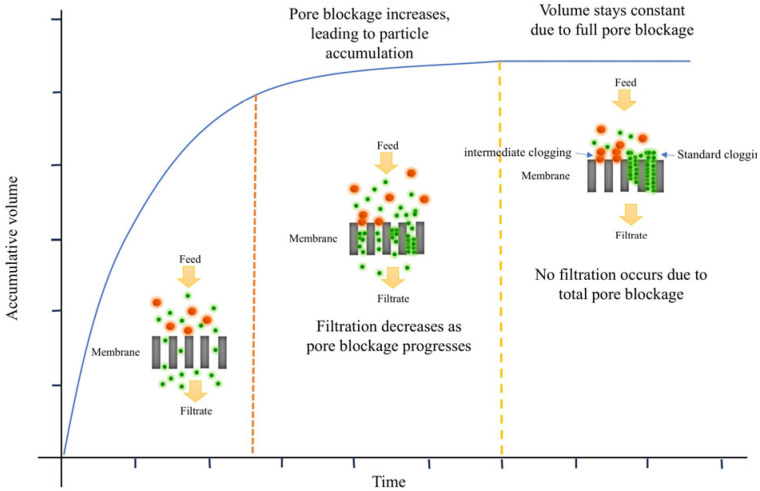
Schematic representation of membrane clogging: dynamic progression and structural changes.

Table S6 in the Supplementary Data presents the adjustment parameters (a, b, and m) of Model 6, as well as the clogging index (*N*), the clogging constant (*k*), and the coefficient of determination (*R*^2^) of Equation (3). Specifically. The clogging index (*N*) for solution BSA at different pressures is close to 1.5, implying the presence of standard clogging (*N* = 1.5028 at *P* = 2 psi, *N* = 1.4976 at *P* = 5 psi, *N* = 1.4975 at *P* = 10 psi, and *N* = 1.5014 at *P* = 20 psi). The clogging index values (*N*) for solution pQR150 are *N* = 1.3470 at *P* = 5 psi and *N* = 1.4237 at *P* = 8 psi, while for solution pGEc47, they are *N* = 1.4417 at *P* = 5 psi and *N* = 1.3147 at *P* = 8 psi. Clogging index (*N*) values between 1 and 1.5 indicate intermediate and standard clogging. These results align with the findings in [18,26,27]. The identification of standard and intermediate clogging mechanisms highlights the physical interactions governing filtration. The standard blocking model is the most accepted theory, where the retained particles are smaller than the average pore size [20]. Standard clogging occurs when particles infiltrate membrane pores, adhering to their walls and progressively reducing permeability [36]. This is evident in BSA solutions, where clogging index values around 1.5 indicate pore narrowing as the dominant mechanism, driven by hydrodynamic transport [37], van der Waals forces [36], and electrostatic interactions. Intermediate clogging, acting as a transition to cake filtration, involves both partial pore blockage and surface accumulation. Clogging index values between 1 and 1.5 for pQR150 and pGEc47 solutions suggest that particle–particle interactions significantly influence this process. Factors such as particle size distribution, surface charge, and fluid velocity determine whether particles remain suspended, deposit on the membrane surface, or penetrate its structure. These findings suggest that membrane clogging can result from pore penetration and surface deposition depending on the solution composition and operating conditions. However, this may not apply to all cases, as clogging behavior varies with system-specific factors. Figure 5 illustrates clogging mechanisms over time, providing a visual representation of pore blockage progression and its effect on filtration.

To further evaluate the models, we compared them with Hermia’s standard and combined models. In this section, we perform a comparative analysis between our proposed models and two models from the literature, applied to the same clogging mechanism for DB01 and DB02, using the Relative Model Fitting Error (*R*^2^ and SSR). The first model, Hermia’s model, represents a standard clogging mechanism. It describes gradual pore constriction due to internal fouling as particles obstruct the membrane pores [27,38]. The second model captures a combined mechanism, transitioning from intermediate clogging—where particles deposit on the membrane surface—to standard clogging through pore constriction. This approach provides a more comprehensive representation of complex fouling transitions [24,27]. The equations for these models can be found in the Supplementary Data, Table S7. When compared to the reference models, the proposed empirical model demonstrated a significantly enhanced ability to predict volume accumulation under the tested clogging mechanisms. The results, summarized in Table 3, highlight the proposed model’s superior predictive performance. It better captures the dynamics of material accumulation and provides more accurate volume predictions than the reference models.

## 4. Conclusions and Perspectives

This work aimed to propose a new empirical fractional estimation approach to model the clogging of membrane filtration. The research aimed to assess the efficacy of eight proposed empirical fractional estimation models for characterizing the clogging of membrane filtration. The proposed empirical fractional estimation models exhibited a strong fit to the data and demonstrated excellent performance under different pressures and filtration solutions, confirming their robustness, reliability, and efficiency. Additionally, the fifth model proved to be the most efficient, achieving a superior fit and the lowest errors compared to the other proposed models across various pressure and solution conditions. Its enhanced predictive capability stems from its ability to accommodate more parameters, which refine its adaptability in representing clogging variations, and from its consideration of the nonlinear dynamics governing the clogging process. These factors collectively reinforce the model’s robustness in describing membrane filtration behavior under diverse conditions.

An analysis of the clogging of the membrane filtration mechanism using the equation of Hermia revealed standard clogging for the first database at different pressures, with the clogging index (*N*) approaching 1.5. In contrast, the second database exhibits a combination of intermediate and standard clogging, with the clogging index (*N*) ranging from 1 to 1.5. Our results are consistent with the conclusions presented in [18,26,27].

The proposed empirical models demonstrated strong performance in simulating and predicting clogging dynamics. However, they are primarily designed for predictive purposes and do not directly identify the underlying mechanisms of clogging. This limitation highlights the importance of future research aiming to integrate physically based parameters into empirical models to enhance their explanatory power regarding clogging mechanisms. For further evaluation of our models, we compared their effectiveness in predicting cumulative volume with a classical model and a combined model from the literature. Our model exhibited better predictive performance compared to the other models while being less explanatory. To further improve model reliability, future studies should focus on expanding the dataset by conducting a more comprehensive literature review and exploring simulation approaches to generate diverse experimental conditions. Additionally, a more detailed classification of datasets according to specific clogging mechanisms—such as standard, intermediate, or combined clogging—would be valuable for gaining deeper insights into the associated physical processes. Such classifications could form the basis for developing hybrid models that combine empirical precision with mechanistic interpretation, offering a more comprehensive understanding of clogging behavior. While the proposed models represent a significant advancement in accurately predicting clogging dynamics, further research is needed to improve their physical interpretability. Strengthening this aspect would not only provide a clearer understanding of the mechanisms driving membrane filtration clogging but also facilitate the development of more robust and versatile predictive tools for various filtration applications.

## Figures and Tables

**Table 1:** The proposed empirical fractional estimation models.

N° Models Proposed	Equation	Boundary Conditions
Model 1	$v=\frac{a\cdot t^{n}}{1+b\cdot t^{m}}$	$v\left(t=0\right)=0,v\left(t\to \infty \right)=\frac{a}{b}\to v_{\infty }(*)$
Model 2	$v=v_{\infty }\left(\frac{a\cdot t^{n}}{1+a\cdot t^{n}}\right)$	v(t=0)=0, $v\left(t\to \infty \right)=v_{\infty }$
Model 3	$v=v_{\infty }\left(\frac{t^{n}}{a+t^{n}}\right)$	v(t=0)=0, $v\left(t\to \infty \right)=v_{\infty }$
Model 4	$v=a\cdot v_{\infty }{\left(\frac{t^{n}}{1+t^{n}}\right)}^{m}$	v(t=0)=0, $v\left(t\to \infty \right)={a\cdot v}_{m}\to v_{\infty }(*)$
Model 5	$v=d{\left(\frac{{a\cdot t}^{n}}{c+b\cdot t^{n}}\right)}^{m}$	v(t=0)=0, $v\left(t\to \infty \right)\to v_{\infty }(*)$
Model 6	$v=\frac{a\cdot t^{n}}{1+b\cdot t^{n}}$	v(t=0)=0, $v\left(t\to \infty \right)=\frac{a}{b}\to v_{\infty }(*)$
Model 7	$v=v_{\infty }\left(\frac{1-e^{-a\cdot t}}{1+c\left(1-e^{-b\cdot t}\right)}\right)$	v(t=0)=0, $v\left(t\to \infty \right)=v_{\infty }{\cdot K\to v}_{\infty }(*)$
Model 8	$v=v_{\infty }\left(\frac{1-e^{-a\cdot t^{n}}}{1+c\left(1-e^{-b\cdot t^{n}}\right)}\right)$	v(t=0)=0, $v\left(t\to \infty \right)=v_{m}\cdot K\to v_{\infty }(*)$

(∗) Indicates the possibility that v  can approach $v_{\infty }$ as t→∞. K: constant. v: accumulated volume; $v_{\infty }:$ maximal accumulated volume; t: time of filtration; $t_{\infty }:$ maximal time extrapolation of filtration corresponding to $v_{\infty }$; a, b, c, d, n, and m: unknown parameters.

**Table 2:** The equations of model 6, its derivatives, and the equation of Hermia.

Model 6 (v = f(t))	Model 6 (t = f(v))	First Derivate	Second Derivate	Equation of Hermia
$v=\frac{a\;t^{n}}{\left(1+b\cdot t^{n}\right)}$	$t=\frac{v^{m}}{{\left(a-b\cdot v\right)}^{m}}$	$\frac{dt}{dv}=\frac{a\cdot m\cdot v^{m-1}}{{\left(a-b\cdot v\right)}^{m+1}}$	$\frac{d^{2}t}{{dv}^{2}}=\frac{a\cdot m\;\left(a\cdot m-a+2\cdot b\cdot v\right)}{{\left(a-b\cdot v\right)}^{m+2}}$	$\frac{d^{2}t}{{dv}^{2}}=k{\left(\frac{dt}{dv}\right)}^{N}$

*v*: accumulated volume; *t*: time of filtration. $m=\frac{1}{n}$ (*m* and *n* constant).

**Table 3:** Results of the comparative analysis of model accuracy.

Solution	Pressure (psi)	Relative Model Fitting Error	Reference Model		Proposed Model							
			Standard Model	Combined Model	Model 1	Model 2	Model 3	Model 4	Model 5	Model 6	Model 7	Model 8
SBA	2	*R* ^2^	0.9927	0.9934	0.9945	0.9936	0.9936	0.9935	0.9937	0.9936	0.9631	0.9897
		SSR	4.23 × 10^6^	3.80 × 10^6^	0.0318	0.0372	0.0372	0.0372	0.0361	0.0372	0.1846	0.0600
	5	*R* ^2^	0.9964	0.9964	0.9981	0.9975	0.9975	0.9944	0.9986	0.9975	0.9920	0.9978
		SSR	1.55 × 10^7^	1.55 × 10^7^	0.0845	0.1096	0.1096	0.2221	0.0611	0.1096	0.2440	0.0892
	10	*R* ^2^	0.9977	0.9977	0.9993	0.9989	0.9989	0.9962	0.9996	0.9989	0.9942	0.9989
		SSR	5.29 × 10^7^	5.29 × 10^7^	0.1571	0.2624	0.2624	0.8774	0.1000	0.2624	0.1000	0.2624
	20	*R* ^2^	0.9971	0.9978	0.9978	0.9978	0.9978	0.9972	0.9978	0.9977	0.9978	0.9964
		SSR	8.47 × 10^7^	6.60 × 10^7^	0.6542	0.6546	0.6546	0.8255	0.6538	0.7152	0.7152	1.0685
pGEc45 (56 kb)	5	*R* ^2^	0.9963	0.9989	0.9988	0.9985	0.9985	0.9988	0.9988	0.9985	0.9991	0.9994
		SSR	0.3914	0.1211	0.0605	0.0652	0.0565	0.0518	0.0924	0.0652	0.0719	0.0887
	8	*R* ^2^	0.9960	0.9968	0.9987	0.9986	0.9986	0.9987	0.9987	0.9986	0.9986	0.9985
		SSR	0.3270	0.2606	0.1275	0.1745	0.1052	0.1190	0.1745	0.0654	0.0757	0.0677
pGEc45 (56 kb)	5	*R* ^2^	0.9989	0.9993	0.9997	0.9997	0.9997	0.9995	0.9997	0.9997	0.9997	0.9997
		SSR	0.2119	0.1402	0.1313	0.1415	0.1258	0.1323	0.1571	0.0748	0.0914	0.0685
	8	*R* ^2^	0.9920	0.9981	0.999	0.9987	0.9987	0.9991	0.9992	0.9987	0.9994	0.9995
		SSR	1.0634	0.2575	0.1070	0.1115	0.1041	0.1204	0.1115	0.1211	0.1109	0.1064

## Data Availability

The data are available upon request.

## Supplementary Materials

The following supporting information can be downloaded at: https://www.mdpi.com/article/10.3390/membranes15040099/s1, Figure S1: Regression plot comparing the calculated vs. experimental volume values for the eight proposed models at different filtration pressures: (a) *P* = 2 psi, (b) *P* = 5 psi, (c) *P* = 10 psi, and (d) *P* = 20 psi for DB01, and (e) *P* = 5 psi (pQR150), (f) *P* = 8 psi (pQR150), (g) *P* = 5 psi (pGEc47), and (h) *P* = 5 psi (pGEc47) for DB02; Table S1: The units of parameters in the proposed empirical fractional estimation models; Table S2: Database statistical analysis; Table S3: Database size and different pressures used in this work; Table S4: Adjustment parameters and error values of the proposed empirical fractional estimation models for membrane filtration clogging at different pressures of the two databases (DB01 and DB02); Table S5: Linear regression vectors [Linear Equation: $y_{i,cal}={\alpha y}_{i,exp}+\beta$] and the performance metrics (R, *R*^2^, and nRMSE) of empirical fractional estimation models; Table S6: Adjustment parameters (a, b, and m) of Model 6, with clogging index (*N*), clogging constant (*k*), and coefficient of determination (*R*^2^) of Equation 3; Table S7: Equations of Hermia’s model.

## Glossary

- Clogging Index (*N*): A value derived from the Hermia equation used to characterize the degree of clogging of membrane filtration.
- Empirical Fractional Estimation Model: A model created using experimental data based on volume changes during membrane clogging.
- Dragonfly Algorithm (DA-nlinfit): An optimization algorithm coupled with nonlinear regression used to predict membrane filtration performance.

## Abbreviations

BSA	Bovine serum albumin
BD	Database
Da	Dragonfly algorithm
N	Clogging index
nMAE	Relative mean absolute error
nRMSE	Relative root mean squared error
pGEc47	Tetracycline resistance 56 kb
pQR150	Kanamycin resistance 20 kb
P	Pressure (psi)
R	Coefficient of correlation
*R* ^2^	Coefficient of determination
$t_{m}$	Time of filtration maximal optimal (min)
$t_{\infty }$	Time of filtration infinite optimal (min)
V	Volume accumulation (m^3^)
$v_{\infty }$	Volume accumulation infinite optimal (m^3^)
$v_{m}$	Volume accumulation maximal optimal (m^3^)
VAF	Variance accounted for metric
Var	Variance
