# Supplementary material for: A Novel Empirical Fractional Approach for Modeling the Clogging of Membrane Filtration During Protein Microfiltration
Source: Membranes (Basel). 2025 Mar 26;15(4):99. doi: 10.3390/membranes15040099 (PMC12029217; doi:10.3390/membranes15040099)
Supplement: Supplementary file 1 [file membranes-15-00099-s001.zip › membranes-3502966-supplementary.pdf]

Supplementary Materials:

A Novel Empirical Fractional Approach for Modeling the Clogging of Membrane Filtration During Protein Microfiltration

Leila Cherifi [1,*], Yamina Ammi [1], Salah Hanini [1], Mohamed Hentabli [1,2,3], Ouafa Belkacem [1] and Jérôme Harmand [4]

1 Laboratory of Biomaterials and Transport Phenomena (LBMPT), Faculty of Technology, University Yahia Fares of Medea, Medea 26000, Algeria; ammi.yamina@yahoo.fr (Y.A.); hanini.salah@univ-medea.dz (S.H.); hentabli.mohamed@univ-medea.dz (M.H.); belkacem.ouafa@univ-medea.dz (O.B.)
2 Process Engineering Department, Faculty of Technology, Hassiba Ben Bouali University of Chlef, Hay Essalem, P.O. Box 151, Chlef, 02000, Algeria
3 Laboratory of Vegetal Chemistry-Water-Energy, Hassiba Ben Bouali University of Chlef, Hay Essalem, P.O. Box 151, Chlef 02000, Algeria
4 Laboratory of Environmental Biotechnology (LBE), National Research Institute for Agriculture, Food and the Environment (INRAE), UR0050, University of Montpellier, 11100 Narbonne, France; jerome.harmand@inrae.fr
* Correspondence: cherifi.leila@univ-medea.dz

Table S1 presents the units of parameters in each proposed empirical fractional estimation model

Table S1. The units of parameters in the proposed empirical fractional estimation models.

Model	a	b	c	d	v_m	t_m
Model 1	$[v].[t]^{-n}$	$[t]^{-m}$	-	-	-	-
Model 2	$[t]^{-n}$	-	-	-	$[v]$	-
Model 3	$[t]^{-n}$	-	-	-	$[v]$	-
Model 4	-	-	-	-	$[v]$	-
Model 5	$[t]^{-n}$	$[t]^{-n}$	-	$[v]$	-	-
Model 6	$[v].[t]^{-n}$	$[t]^{-n}$	-	-	-	-
Model 7	$[t]^{-1}$	$[t]^{-1}$	-	-	$[v]$	-
Model 8	$[t]^{-n}$	$[t]^{-n}$	-	-	-	-

- [t]: unite of time. [v]: unite of volume.

Table S2 shows a statistical analysis of database variables.

Table S2. Database Statistical Analysis.

Databases	Solution	Pressures (psi)	Variable	Min	Max	Mean	STD
DB 01	BSA	P= 2	$t(min)$	0.3799	86.6097	37.0694	27.1393
			$v(m^3)$	726.6440	10173.0000	7778.2459	2251.0000
		P= 5	$t(min)$	0.3799	85.0902	33.3203	26.5737
			$v(m^3)$	1937.7200	22283.7000	16709.8511	6142.7000
		P= 10	$t(min)$	0.0000	108.6420	43.4582	37.5477
			$v(m^3)$	1211.0700	51833.9000	38064.5412	15885.0000
		P= 20	$t(min)$	0.3788	81.0607	25.6379	25.6661
			$v(m^3)$	4117.6500	63944.6000	43293.8061	18128.0000
DB 02	pQR150	P= 5	$t(min)$	0.0000	35.0000	11.5195	9.4282
			$v(10^{-6}m^3)$	0.0000	4.2216	2.3813	1.5266
		P= 8	$t(min)$	0.0000	35.0000	9.1865	7.4096
			$v(10^{-6}m^3)$	0.0000	4.6639	2.9845	1.6623
	pGEc47	P= 5	$t(min)$	0.0000	35.0000	10.0541	8.8337
			$v(10^{-6}m^3)$	0.0000	6.6177	3.8831	2.4134
		P= 8	$t(min)$	0.0000	35.0000	10.0298	8.8963
			$v(10^{-6}m^3)$	0.0000	6.2080	4.0005	2.3466

Table S3 summarizes the sizes and pressure conditions of the database.

Table S3. Database size and different pressures are used in this work.

	DB 01				DB 02			
	BSA				pQR150		pGEc47	
Pressure (psi)	2	5	10	20	5	8	5	8
Database size	115	116	92	89	48	41	40	40

Table S4. Adjustment parameters and error values of the proposed empirical fractional estimation models for membrane filtration clogging at different pressures of the two databases (DB 01 and DB 02).

Model	Parameter<br>Pressure (psi)	a	b	c	d	n	m	$v_\infty(10^4.m^3)$	R^2	nRMSE %	nMAE %	nchi %	SEP%	VAF%
							DB 01							
Model 1	p= 2	0,1625	0,2262	-	-	1,1459	1,0644	-	0,9945	2,1539	1,7387	0,0372	2,1527	99.4492
	p= 5	0.3148	0.0897	-	-	0.9553	1.0375	-	0.9981	1,6312	1,2143	0,0456	1,6297	99.8053
	p= 10	0.6137	0.0770	-	-	0.9776	1.0509	-	0.9993	1,0860	0,8073	0,0473	1,0968	99.9316
	p= 20	1.2827	0.1799	-	-	0.9193	0.9232	-	0.9978	1,9596	1,2199	0,1746	1,9924	99.7770
Model 2	p= 2	0.1627	-	-	-	0.9107	-	1,0904	0.9936	2,3342	1,9322	0,0431	2,3343	99.3558
	p= 5	0.1254	-	-	-	1.1091	-	2.3470	0.9975	1,8562	1,2818	0,0586	1,8531	99.7475
	p= 10	0.1022	-	-	-	1.1232	-	5.4805	0.9989	1,4055	1,0344	0,0763	1,4181	99.8857
	p= 20	0,1836	-	-	-	0,9261	-	6.9917	0.9978	1,9597	1,2199	0,1723	1,9924	99.7769
model3	p= 2	6,1474		-	-	0.9107	-	1.0904	0.9936	2,3342	1,9322	0,0431	2,3343	99.3558
	p= 5	7.9741		-	-	1.1091	-	2.3470	0.9975	1,8562	1,2818	0,0586	1,8531	99.7475
	p= 10	9.7815	-	-	-	1.1232	-	5.4805	0.9989	1,4055	1,0344	0,0763	1,4181	99.8857
	p= 20	6.9917	-	-	-	0.9261	-	5.4463	0.9978	1,9597	0,1723	0,1723	1,9924	99.7769
Model 4	p=2	3.0194	-	-	-	0.6540	3.1373	0.3878	0.9935	2,3353	1,8942	0,0436	2,3343	99.3540
	p= 5	6.9427	-	-	-	0.7747	3.5101	0.3607	0.9944	2,7777	1,9910	0,1331	2,7706	99.4377
	p= 10	3.2078	-	-	-	0.7734	3.9047	1.8188	0.9962	2,5748	2,0319	0,2635	2,5946	99.6179
	p= 20	5.0704	-	-	-	0,6521	2,8784	1,4920	0.9972	2,2021	1,5700	0,2207	2,2377	99.7186
Model 5	p= 2	0.2909	0.2626	0.7361	0.9602	0.7798	1.5450	-	0.9937	2,2959	1,8938	0,0430	2,2954	99.3744
	p= 5	1.3370	1.8824	101.3699	2.6672	1.5633	0.4960	-	0.9986	1,3883	1,0382	0,0335	1,3883	99.8591
	p= 10	8.7545	9.6073	499.2764	5.5817	1.4998	0.5452	-	0.9996	0,8674	0,6229	0,0315	0,8764	99.9565
	p= 20	11.7122	5.5020	33,0878	3,3907	0.9468	0.9524	-	0.9978	1,9573	1,2129	0,1792	1,9901	99.7771
Model 6	p= 2	0,1774	0.1627	-	-	0.9107	-	-	0.9936	2,3342	1,9322	0,0431	2,3343	99.3558
	p= 5	0.2943	0.1254	-	-	1.1091		-	0.9975	1,8562	1,2818	0,0586	1,8531	99.7475
	p= 10	0.5603	0.1022	-	-	1.1232		-	0.9989	1,4055	1,0344	0,0763	1,4181	99.8857
	p= 20	1.2838	0.1836	-	-	0.9261		-	0.9978	1,9597	1,2199	0,1723	1,9924	99.7769
Model 7	p= 2	$7.9676.10^{-8}$	0,0003	$8.0392.10^{4}$	-	-	-	6,4527	0.9936	2,3344	1,9323	0,0431	2,3343	99.3558
	p= 5	0.0421	0.0404	2.4367	-	-	-	7.5687	0.9979	1,6737	1,2447	0,0481	1,6720	99.7943
	p= 10	0.0362	0.0348	2.3530	-	-	-	17.3622	0.9993	1,1359	0,8730	0,0500	1,1473	99.9253
	p= 20	0.0340	0.0391	4.5158	-	-	-	36.4044	0.9976	2,0486	1,2692	0,1931	2,0812	99.7562
Model 8	p= 2	0.2040	133.0174	0.8961	-	0.6691		1.8866	0.9896	2,9540	2,5005	0,0707	2,9567	98.9606
	p= 5	0.1818	16.4385	0.0163	-	0.7549		2.2345	0.9965	2,1880	1,6395	0,0844	2,1911	99.6473
	p= 10	0.0356	17.6342	0.0342	-	1.0021		2.4051	0.9993	1,1360	0,8704	0,0526	1,1473	99,9253
	p= 20	0.2094	822.8046	0.0909	-	0.7157		6.9198	0.9964	2,5007	1,8950	0,2891	2,5460	99.6358

Model	Parameter / Pressure (psi)	a	b	c	d	n	m	$v_\infty(10^{-6}.m^3)$	R^2	nRMSE %	nMAE %	nchi %	SEP%	VAF%
	DB 02													
Model 1	p= 5 (150)	1.0998	0.3292	-	-	0.9492	0.8331	-	0.9988	2,1934	1,7278	2,1963	0,1258	99,8774
	p= 8 (150)	2.3280	0.5154	-	-	0.9863	0.9580	-	0.9987	1,7117	1,2964	1,7122	0,0971	99,8690
	p= 5 (47)	2.0627	0.3083	-	-	1.0040	0.9776	-	0.9997	1,0014	0,8032	1,0018	0,0438	99,9680
	p= 8 (47)	3.7735	0.7611	-	-	0,9774	0,8952	-	0,999	1,4119	1,0921	1,4123	0,0875	99,9035
Model 2	p= 5 (150)	0.1708	-	-	-	0,8341	-	5.6352	0.9985	2,3971	2,0241	2,4021	0,1467	99,8533
	p= 8 (150)	0.4334	-	-	-	0.9294	-	5.0664	0.9986	1,7481	1,3161	1,7491	0,0971	99,8635
	p= 5 (47)	0.2680	-	-	-	0,9627	-	7.4703	0,9997	1,0397	0,8081	1,0404	0,0463	99,9655
	p= 8 (47)	0.4291	-	-	-	0.8005	-	7.0848	0,9987	1,6508	1,2562	1,6523	0,1174	99,8679
Model 3	p= 5 (150)	5.8551	-	-	-	0.8341	-	5.6352	0,9985	2,3971	2,0241	2,4021	0,1467	99,8533
	p= 8 (150)	5.0664	-	-	-	0.9294	-	2.3075	0.9986	1,7481	1,3161	1,7491	0,0971	99,8635
	p= 5 (47)	3.7313	-	-	-	0.9627	-	7.4703	0,9997	1,0397	0,8081	1,0404	0,0463	99,9655
	p= 8 (47)	2.3303	-	-	-	0,8005	-	7.0848	0,9987	1,6508	1,2562	1,6523	0,1174	99,8679
Model 4	P= 5 (150)	1.1757		-	-	0.4962	3.1138	6.0713	0.9988	2,1495	1,6751	2,1501	0,1217	99,8825
	p= 8 (150)	0.7917		-	-	0.7533	1.7688	6.6756	0,9987	1,7058	1,3473	1,7055	0,0972	99,8698
	p= 5 (47)	0,877		-	-	0.6924	2.3537	9,2778	0,9995	1,2139	0,9562	1,2129	0,0644	99,9531
	p= 8 (47)	0.8984		-	-	0.6393	1.8040	8.3261	0,9991	1,3594	1,0071	1,3598	0,0825	99,9105
Model 5	p= 5 (150)	7.5302	5.5527	4.8721	2.5372	0.4803	3.4534	-	0,9988	2,1497	1,6626	2,1501	0,1260	99,8826
	p= 8 (150)	1.8784	0,8817	1,2103	1,7957	0.8124	1.4061	-	0.9987	1,6887	1,3067	1,6887	0,1005	99,8725
	p= 5 (47)	4.3364	1.2671	3.1346	1,6109	0.8640	1,2676	-	0,9997	0,9682	0,7776	0,9683	0,0438	99,9701
	p= 8 (47)	6.2844	5,1563	2,1070	3,7263	0.5342	3.7756	-	0.9992	1,2824	0,9924	1,2823	0,0775	99,9204
Model 6	p= 5 (150)	0.9624	0.1708	-	-	0.8341	-	-	0,9985	2,3971	2,0241	2,4021	0,1467	99,8533
	p= 8 (150)	2.1956	0.4334	-	-	0.9294	-	-	0.9986	1,7481	1,3161	1,7491	0,0971	99,8635
	p= 5 (47)	2.0020	0.2680	-	-	0.9627	-	-	0,9997	1,0397	0,8081	1,0404	0,0463	99,9655
	p= 8 (47)	3,0403	0,4291	-	-	0,8005	-	-	0.9987	1,6508	1,2562	1,6523	0,1174	99,8679
Model 7	p= 5 (150)	0.0866	0.1633	1.8471	-	-	-	12.8682	0.9991	1,8282	1,5053	1,8310	0,0881	99,9146
	p= 8 (150)	0.0786	0.0906	5.0377	-	-	-	29.1727	0,9986	1,7424	1,3303	1,7424	0,1005	99,8642
	p= 5 (47)	0.0882	0.1075	2.4076	-	-	-	23.3690	0,9997	0,9523	0,7412	0,9528	0,0386	99,9710
	p= 8 (47)	0.1197	0,1725	3,929	-	-	-	31,1071	0.9994	1,0870	0,7871	1,0874	0,0525	99,9427
Model 8	p= 5 (150)	0.0559	0.1079	3.8352	-	1.1829	-	21.2195	0.9994	1,5879	1,2182	1,5874	0,0672	99,9360
	p= 8 (150)	0.5990	0.1319	-0.3382	-	0.8341	-	3.2295	0,9985	1,8481	1,3660	1,8496	0,1172	99,8469
	p= 5 (47)	0.0884	0.1149	2.4952	-	1.0400	-	23.7414	0,9997	0,9269	0,7184	0,9271	0,0386	99,9726
	p= 8 (47)	0.1032	0.1527	5,0826	-	1,0757	-	38,1018	0.9995	1,0274	0,7974	1,0274	0,0475	99,9488

(150): solution of pQR150, (47): solution of pGEc47.

Figure S1 shows a regression plot comparison of the calculated vs. experimental volume values for eight models proposed at different pressures and solutions filtration: (a) for DB 01 and (b) for DB 02. Each proposed empirical fractional estimation model was applied to all different pressures.

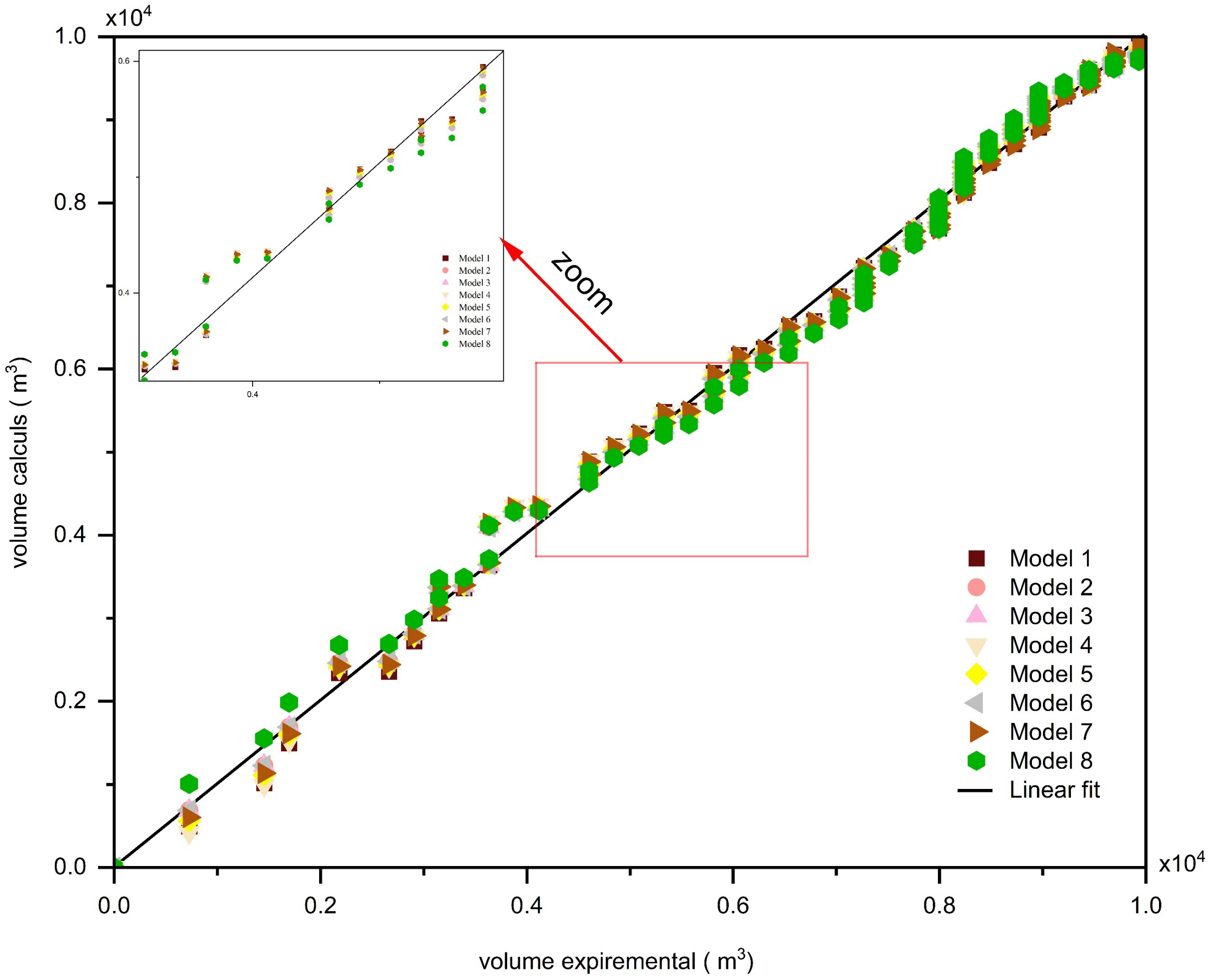

(a)

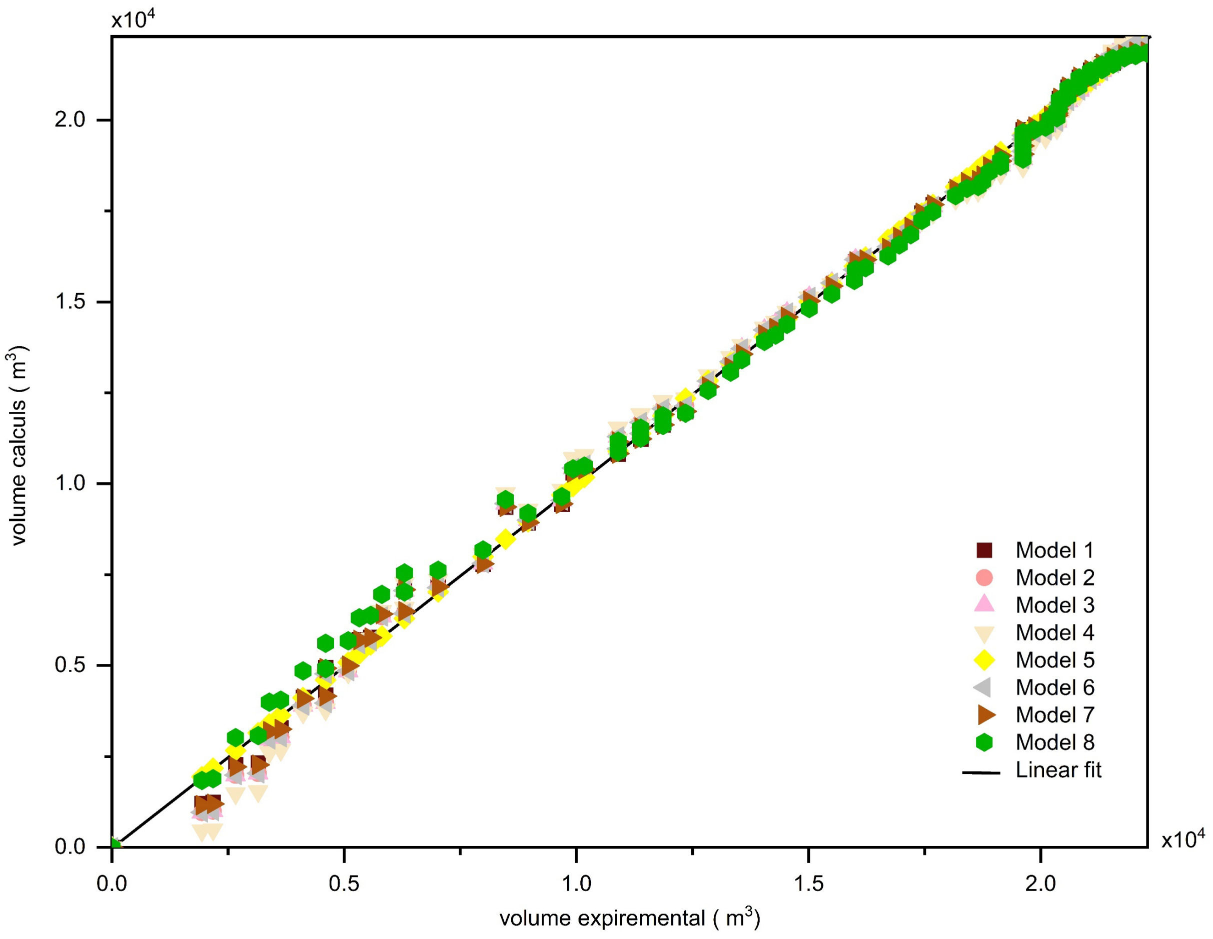

x10^4
2.0
1.5
1.0
0.5
0.0
volume calculs (m^3)
Model 1
Model 2
Model 3
Model 4
Model 5
Model 6
Model 7
Model 8
Linear fit
x10^4
0.0
0.5
1.0
1.5
2.0
volume expiremental (m^3)

(b)

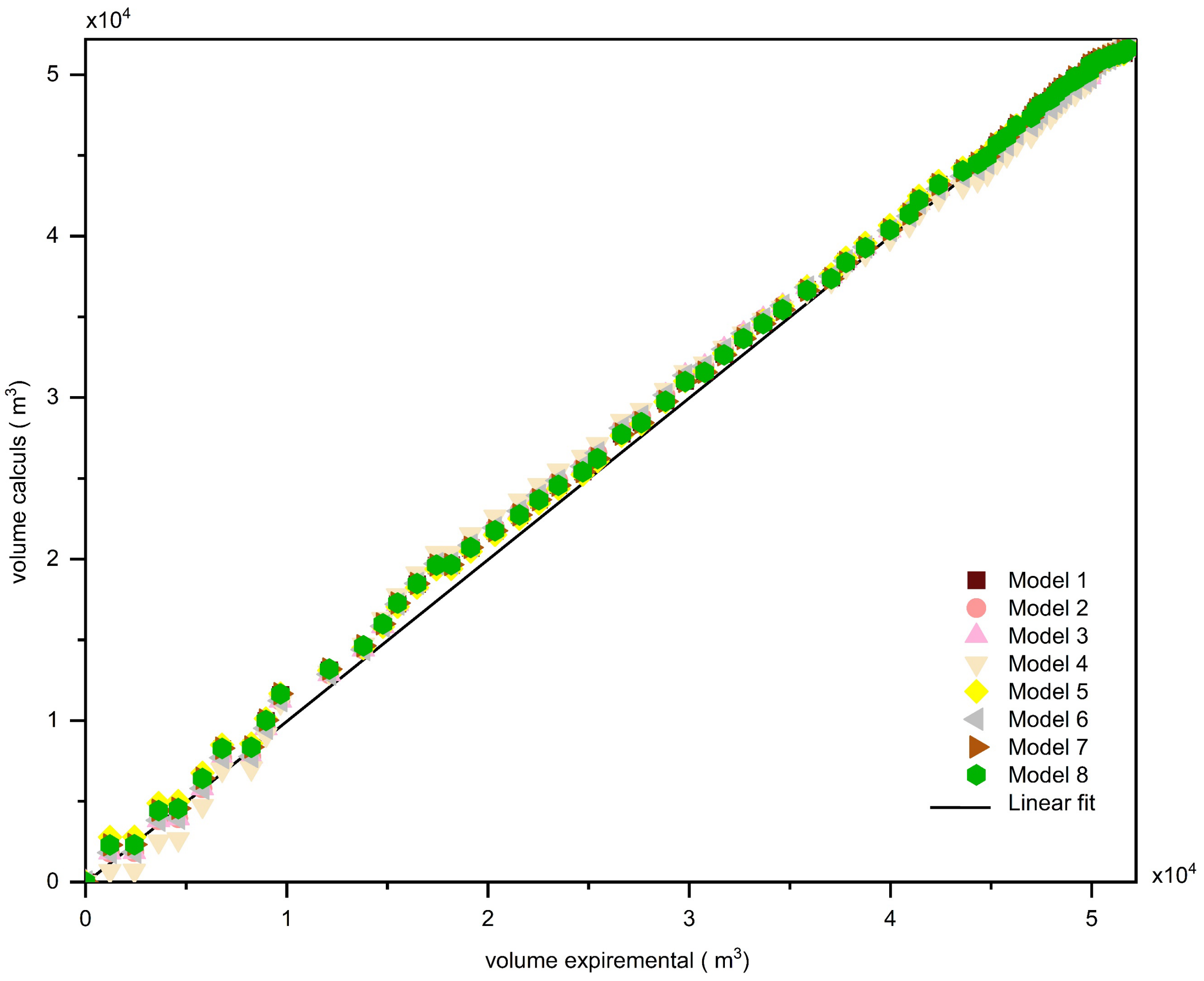
x10^4
5
4
3
2
1
0
volume calculs (m^3)
Model 1
Model 2
Model 3
Model 4
Model 5
Model 6
Model 7
Model 8
Linear fit
x10^4
0
1
2
3
4
5
volume expiremental (m^3)

(c)

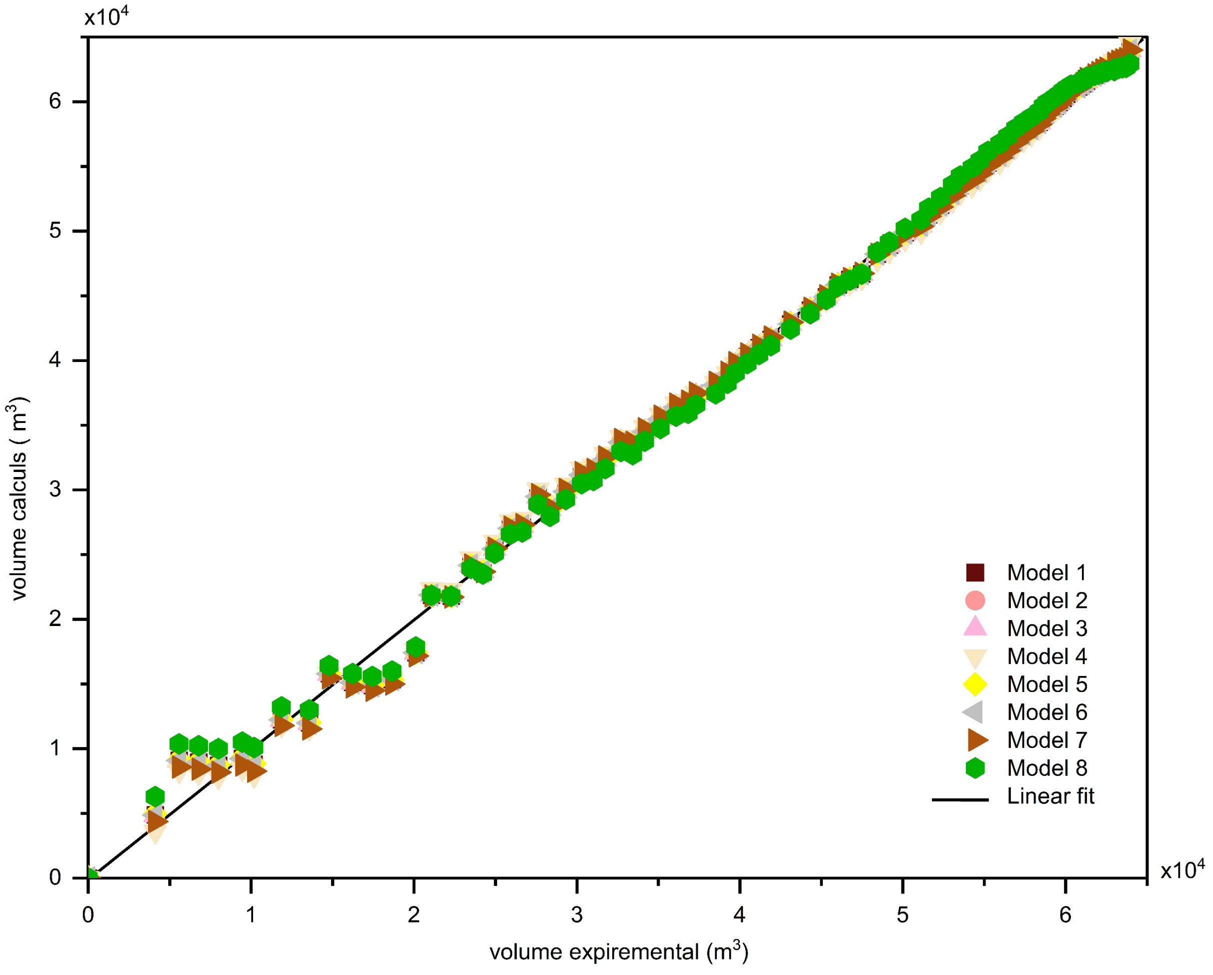
x10^4
6
5
4
3
2
1
0
volume calculs (m^3)
Model 1
Model 2
Model 3
Model 4
Model 5
Model 6
Model 7
Model 8
Linear fit
x10^4
0
1
2
3
4
5
6
volume expiremental (m^3)

(d)

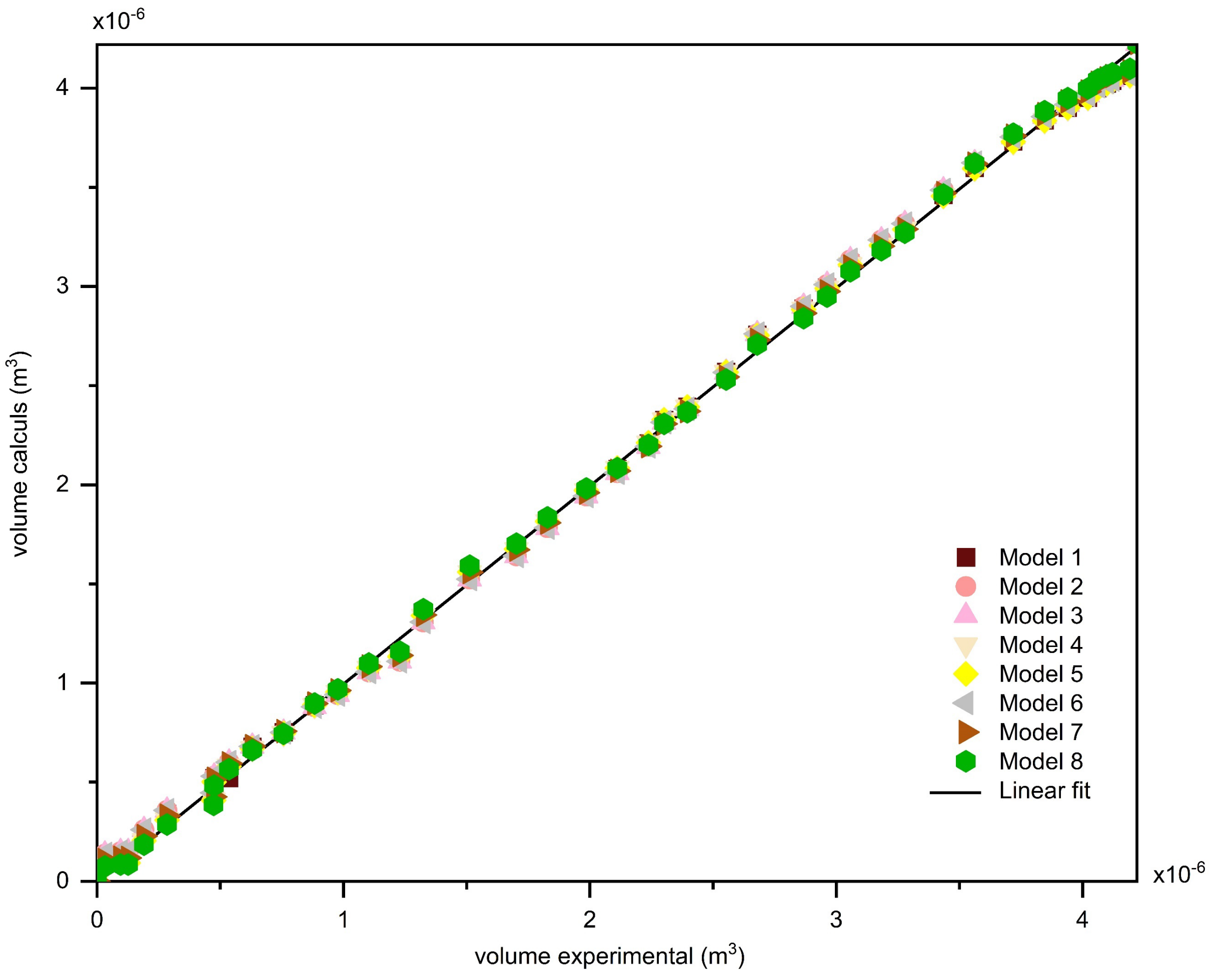
x10^-6
volume calculs (m^3)
volume experimental (m^3)
x10^-6
Model 1
Model 2
Model 3
Model 4
Model 5
Model 6
Model 7
Model 8
Linear fit

(e)

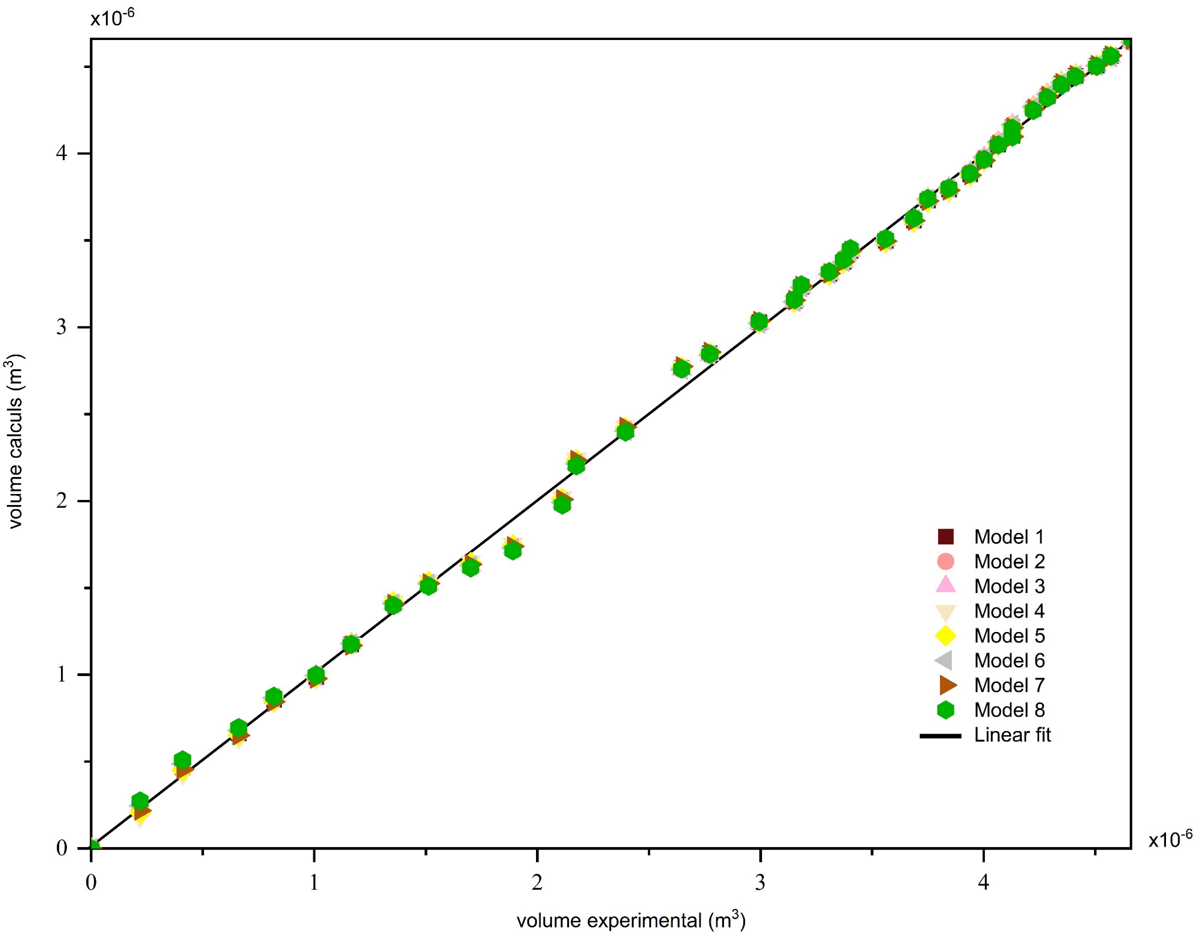

x10-6
volume calculs (m3)
0
1
2
3
4
x10-6
volume experimental (m3)
Model 1
Model 2
Model 3
Model 4
Model 5
Model 6
Model 7
Model 8
Linear fit

(f)

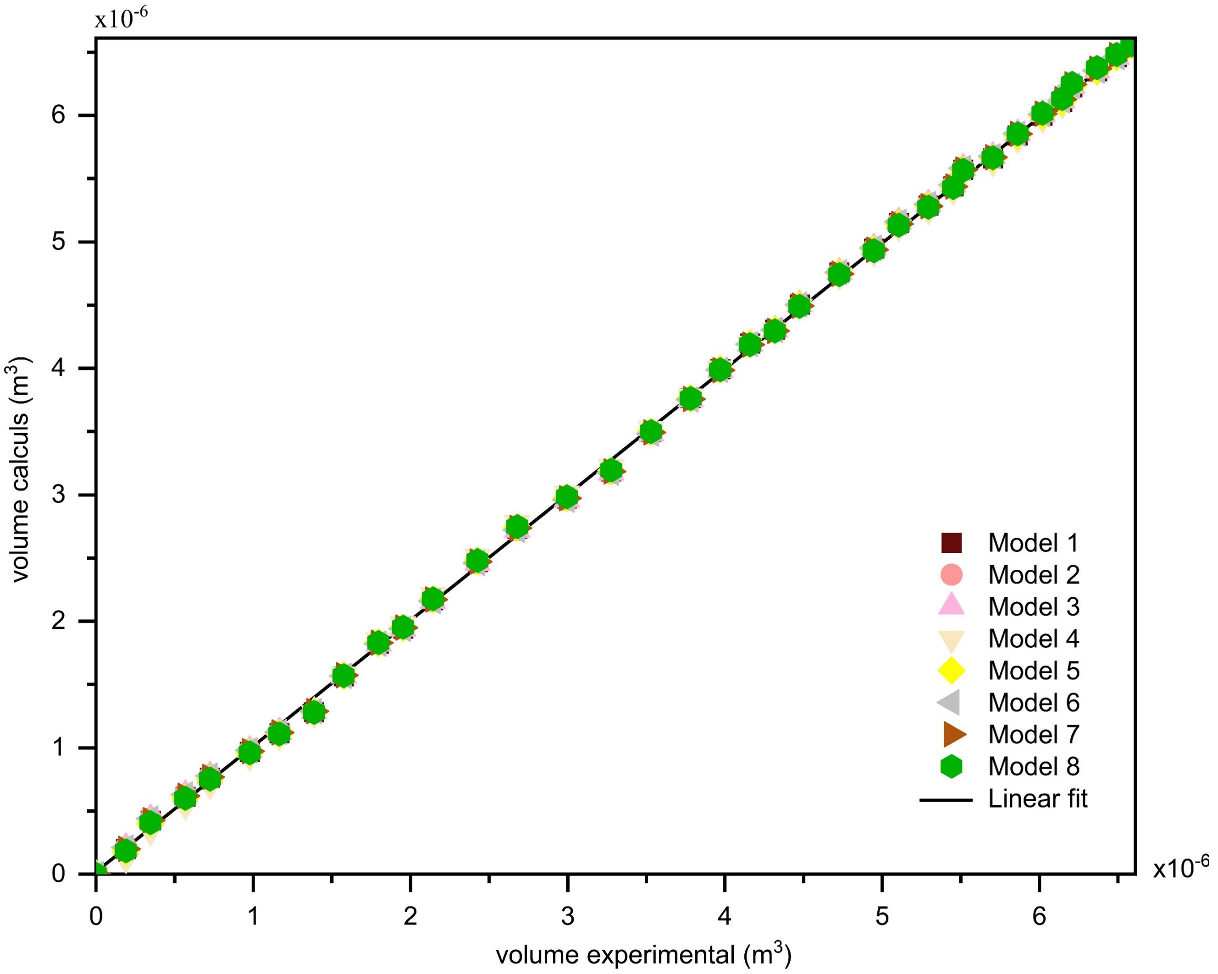
x10-6
6
5
4
3
2
1
0
volume calculs (m3)
Model 1
Model 2
Model 3
Model 4
Model 5
Model 6
Model 7
Model 8
Linear fit
x10-6
0
1
2
3
4
5
6
volume experimental (m3)

(g)

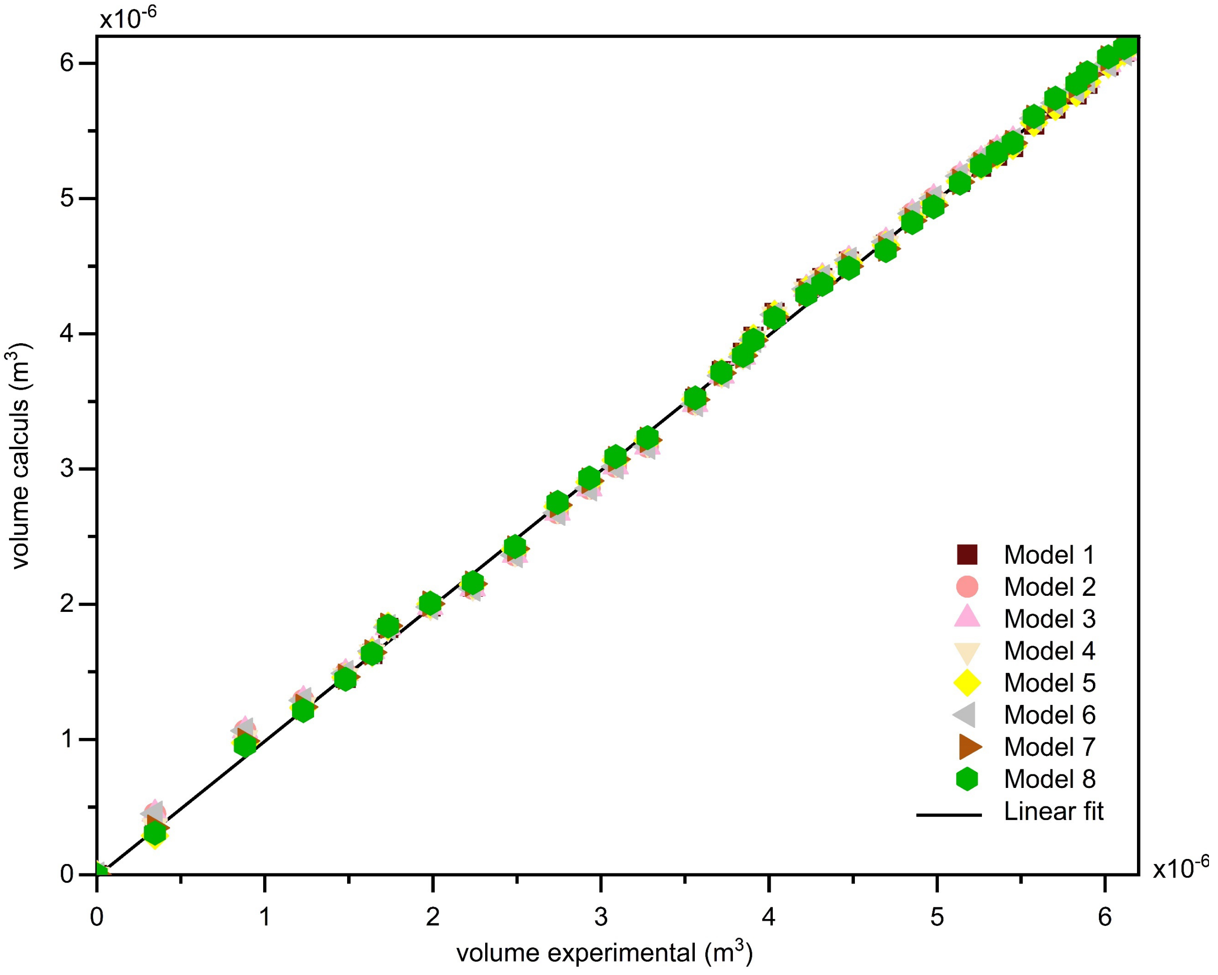

(h)

Figure S1 Regression plot comparing the calculated vs. experimental volume values for the eight proposed models at different filtration pressures: (a) P=2psi, (b) P=5psi, (c) P=10 psi and (d) P= 20 psi for DB01 and (e) P=5 psi (pQR150), (f) P=8 psi (pQR150), (g) P=5 psi (pGEc47) and (h) P=5 psi (pGEc47) for DB02

Table S5 displays the linear regression vector values from the analysis comparing calculated and experimental volume values (figure S1), along with the performance metrics for the eight proposed empirical fractional estimation models.

Table S5. Linear regression vectors [Linear Equation: $y_{i,cal} = \alpha y_{i,exp} + \beta$] and the performances metrics (R, R^2, and nRMSE) of empirical fractional estimation models

Databases	Solution	Pressures (psi)	α	β	R	R^2	nRMSE%
DB01	BSA	P=2	0.9999	10.0000	0.9973	0.9945	2.2748
		P=5	0.9999	-71.0000	0.9988	0.9976	1.8974
		P=10	0.9999	10.0000	0.9993	0.9973	2.3201
		P=20	0.9999	100.0000	0.9987	0.9974	2.2367
DB02	pQR150	P=5	0.9999	$0.0018.10^{-5}$	0.9995	0.9989	2.2384
		P=8	0.9999	$0.0072.10^{-5}$	0.9993	0.9985	1.8578
	pGEc47	P=5	1.0000	$0.0009.10^{-5}$	0.9998	0.9996	1.0907
		P=8	1.0000	$0.0014.10^{-5}$	0.9995	0.9990	1.4092

Table S6. Adjustment parameters (a, b, and m) of Model 6, with clogging index (*N*), clogging constant (k), and coefficient of determination (R^2) of Equation 3

Databases	Solution	Pressures (psi)	a	b	m	*N*	k	R^2
DB 01	BSA	P=2	0.1774	0.1627	1.0981	1.5028	16.3987	0,9858
		P=5	0.2943	0.1254	0.9016	1.4976	8.1043	0,9981
		P=10	0.5603	0.1022	0.8903	1.4975	1.3152	0,9931
		P=20	1.2838	0.1836	1.0798	1.5014	0.0611	0,9991
DB 02	pQR150	P=5	0.9624	0.1708	1.1989	1.3470	0.3508	0.9989
		P=8	2.1956	0.4334	1.0760	1.4273	0.1283	0.9982
	pGEc47	P=5	2.0020	0.2680	1.0387	1.4417	0.1002	0.9981
		P=8	3.0403	0.4291	1.2492	1.3147	0.0629	0.9926

Table S7. Equation of Hermia's model

**Models**	**Equations**	**Fouling parameter**	**reference**
**Standard blocking**	$V = (V_0 . t).\left(1 + t\frac{K_s . V_0}{A_0}\right)^{-1}$	**$K_s(m^{-1})$**	[28, 38]
**Intermediate–standard**	$V = \frac{A_0}{K_{ic}}.ln\left(1 + \frac{2K_{ic}.\left(V_0/A_0\right).t}{2 + K_s\left(V_0/A_0\right).t}\right)$	**$K_{ic}(m^{-1})$ and $K_s(m^{-1})$**	[24, 28]
